# Supplementary material for: Tead1a Initiates Transcriptional Priming Through the TEAD1a/YAP‐Notch1‐Spi1/Cebpα Axis to Promote Neutrophil Fate
Source: Adv Sci (Weinh). 2025 Aug 28;12(41):e05441. doi: 10.1002/advs.202505441 (PMC12591127; doi:10.1002/advs.202505441)
Supplement: Supplementary file 4 — Supplementary Dataset [file ADVS-12-e05441-s004.zip › 3-1.zip!/3-1/02-1.cluster/cluster0/cluster0_vlnplot.pdf]

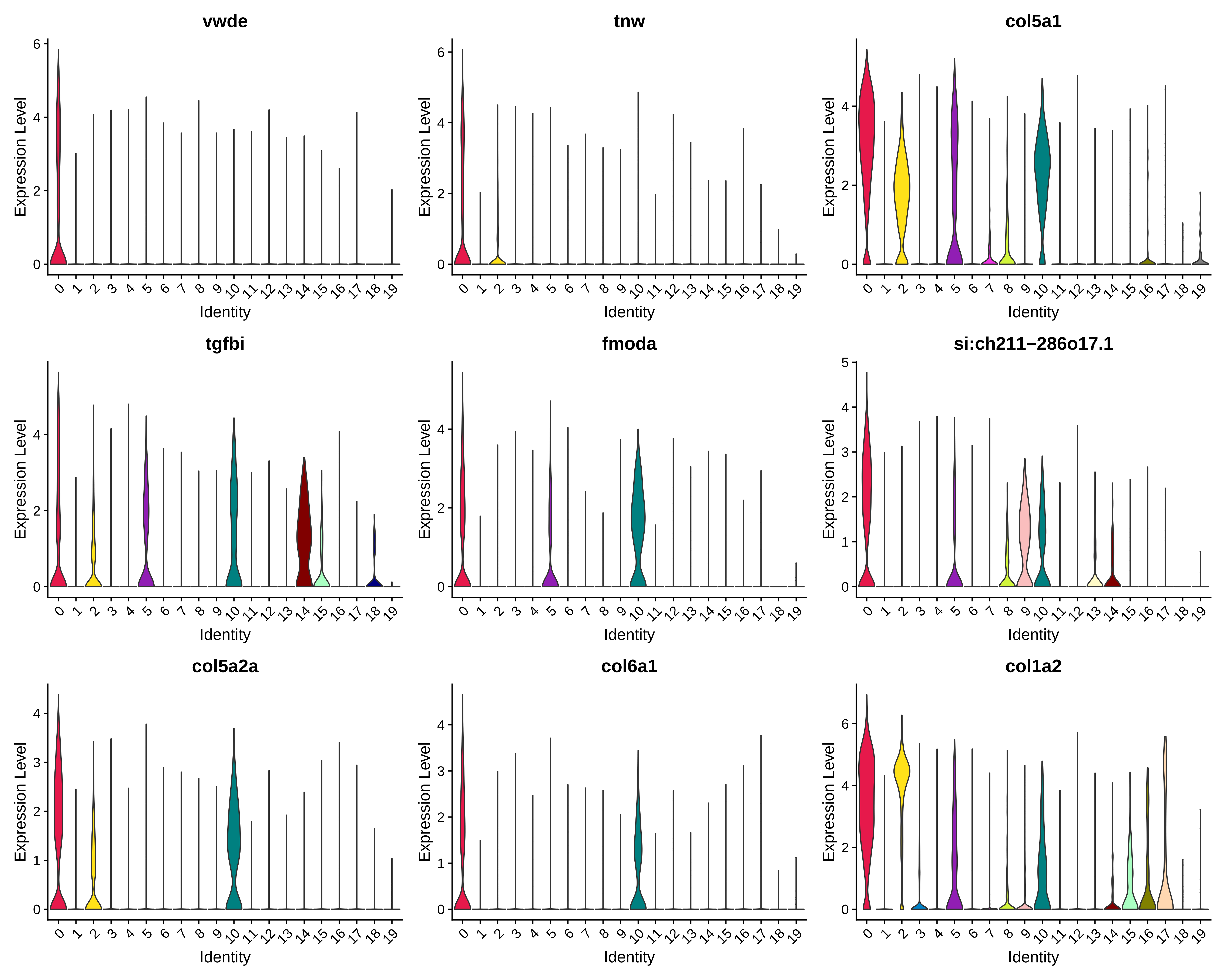
vwde
tnw
col5a1
tgfbi
fmoda
si:ch211−286o17.1
col5a2a
col6a1
col1a2
Expression Level
Identity
0 1 2 3 4 5 6 7 8 9 10 11 12 13 14 15 16 17 18 19
